# A Case of Poisoning by the Seeds of the Datura Stramonium

**Published:** 1847-04

**Authors:** D. G. Gregory

**Affiliations:** Rutersville, Texas


Art. III.—*A Case of Poisoning by the Seeds of the Datura Stramonium.* By Dr. D. G. Gregory, of Rutersville, Texas.

On the morning of the 15th of August last, before I had risen from bed, Mr. H. was at my door requesting me to visit his negro man, who, he said, was laboring under the effects of poison. When I arrived at his house, I found the man in the following condition: Skin a little below the natural temperature, pulse slow and somewhat intermittent, pupils dilated, breathing difficult; patient said he could not distinguish a man from a horse at a distance of twenty paces. I gave him a dose of olive oil, ordered the pediluvium, put a blister on his stomach, and gave him warm brandy toddy. The symptoms soon changed for the better, and he improved in appearance until about 6 o'clock, p. m., when I was hastily summoned to the patient again. His symptoms now had assumed the most alarming character, and it was with the utmost difficulty that I succeeded, by the use of strong stimulants and rubifacients, in restoring the natural warmth. This, however, was done, and being kept up until the next day, the patient was so far restored that no more medicine was required, and he soon recovered.

The history of the case, as related by the patient and his master, was as follows: A few months previously he had been seriously injured in one of his testicles by the falling of a tree, for which he was attended by a physician in the neighborhood, who, called into the service of the United States, left his patient not entirely well. The patient had been told by some travelers that the Jamestown weed would cure him, and they advised him to use the seeds. Accordingly he ate a quantity in the evening, and next morning was taken sick; his master, supposing it was an attack of fever, sent a messenger for a physician, but soon discovered symptoms of poisoning, and commenced giving ipecac. and castor oil largely. Some peculiarities marked the first symptoms: at breakfast he was given a cup of coffee, around which he danced and frolicked until he spilled the coffee and broke the cup. He was next sent to the field to gather and shell a sack of corn; he pulled the corn and placed it in a pile, around which he jumped and danced until he became insensible and began to froth at the mouth and nose. The vomiting and purging had been kept up until I saw him, during which time he discharged a great many seeds.

I learned, about two months ago, that the disease of the testicle had got entirely well. Was it the stramonium that cured him?

Rutersville, Texas, February 15, 1847.

